# Attention-deficit/hyperactivity disorder (ADHD) in adults: a multilayered approach to a serious disorder of inattention to the future

**DOI:** 10.1055/s-0044-1791513

**Published:** 2024-10-02

**Authors:** André Palmini

**Affiliations:** 1 Pontifícia Universidade Católica do Rio Grande do Sul, Escola de Medicina, Núcleo de Neurociências, Porto Alegre RS, Brazil.; 2 Pontifícia Universidade Católica do Rio Grande do Sul, Hospital São Lucas, Serviço de Neurologia, Porto Alegre RS, Brazil.

**Keywords:** Attention Deficit Disorder with Hyperactivity, Adult, Frontal Lobe, Executive Function, Reward, Transtorno do Déficit de Atenção com Hiperatividade, Adulto, Lobo Frontal, Função Executiva, Recompensa

## Abstract

Attention-deficit/hyperactivity disorder (ADHD) affects people of all ages, yet its presentation varies as the person matures and social demands increase from childhood into adulthood. Interestingly, when analyzing the disorder in adults, it is not immediately clear what the ‘attention deficit’ in the ADHD denomination exactly means. Adults with ADHD have a broad range of difficulties, far beyond an attentional deficit, that impact negatively their social functioning and often lead to failures in all walks of life. Therefore, in this review, I attempt to reconcile the notion of attention deficit with the protean manifestations of ADHD in adults through a proposal that ADHD symptoms have as a common denominator an *inattention to the future* . I build this construct through a multilayered approach, progressing from the epidemiological and clinical considerations for Diagnostic and Statistical Manual of Mental Disorders (DSM) diagnosis, to a deeper understanding of the disorder, discussing how these patients fail to anchor the present into the future (i.e., to be attentive to future consequences), thus failing to approximate future goals from present action. Integrating cognitive observations with imaging abnormalities, it is possible to propose that ADHD in adults is perhaps the most prevalent frontal lobe disorder in humans, ultimately impacting upon psychosocial management and treatment strategies.

## Resumo

O transtorno de déficit de atenção/hiperatividade (TDAH) afeta pessoas de todas as idades, embora a apresentação varie de acordo com a maturação cerebral e as demandas sociais, da infância para a idade adulta. Interessantemente, quando se analisa o TDAH em adultos, não fica imediatamente claro o que quer dizer o ‘déficit de atenção’. Adultos com TDAH apresentam um conjunto amplo de dificuldades, que vão muito além de um déficit atencional, impactando negativamente seu funcionamento social e levando a fracassos recorrentes em vários aspectos da vida. Nessa revisão eu tento conciliar a noção de *deficit de atençã* o com as complexas manifestações do TDAH em adultos, através da proposição de que os sintomas têm como denominador comum uma *desatenção ao futuro* . Eu construo esse argumento através de uma abordagem que se inicia com uma revisão dos aspectos clínicos e epidemiológicos até uma compreensão mais profunda do distúrbio, discutindo como esses pacientes têm dificuldade em ancorar o presente no futuro (ou seja, estar atentos às consequências futuras), assim falhando em aproximar objetivos futuros de ações no presente. A partir da integração do contexto cognitivo com alterações de neuroimagem é possível propor que o TDAH em adultos é talvez a forma mais prevalente de doença de lobo frontal em humanos, com impacto no manejo psicossocial e na busca de estratégias terapêuticas.

## INTRODUCTION

### A brief promenade through executive functions and the reasons for a multilayered approach to ADHD in adults

Within a biopsychosocial perspective, the notion of health or feeling well is inseparable from the capacity to seek satisfaction of social needs that will allow individuals to fully integrate in their environment. By social needs I mean those that are not immediately related to basic survival instincts, yet once fulfilled, assure a good quality of life. The organization of behavior according to social needs requires a set of abilities that begins with the reckoning of such needs and progress with systematic decision making toward accomplishing goals, while at the same time monitoring and controlling impulses that satisfy immediate needs or bring immediate rewards but often compromise future achievements in the personal and social spheres.

The evolution of the human brain, mostly because of the need to navigate in increasingly complex societies, led to the development of structures and circuits that allow ongoing identification of priorities and the organization of behaviors targeted to attend to those priorities. Neurocognitive functions allowing the complex regulation of behavior toward defined goals, integrating past experiences with anticipation of future consequences, while at the same time modulating emotional responses, are known collectively as executive functions. 1 Pragmatically speaking, executive functions relate to the efficacy of daily functioning, and adults with attention-deficit/hyperactivity disorder (ADHD) often function poorly in many demands of the day.

Executive functions can be disentangled into four integrated modules. The first is the ability to select and maintain attentional focus to the priority of each moment or circumstance and avoid interferences that deviate the individual from such attentional priority. Because keeping focused on priority tasks often collide with the tendency to divert attention and energy toward more pleasurable attractions, the brain must anchor the present into the future and provide significant salience to the (future) positive consequences of controlling the impulse to depart from priority to pleasure. Because the future in fact does not exist, such anchoring of the palpable present into the nonpalpable future depends upon a *representation of the future as if it were concrete* in circuits centered in the prefrontal and dorsolateral frontal cortices. 2 3 4 Thus, *attention* , in this context, means attention to the future or to the future consequences of acts done (or not done) today. As we will see, the real problem of adults with ADHD centers around an *inattention to the future* , which, in my view, encompasses the multifaceted manifestations of executive dysfunction, leading sometimes to diagnostic confusion and significant treatment challenges.

The second module involves the ability to retrieve relevant memories to the task or decision at hand and guide behavior according to the outcome of previous actions in similar contexts, which are kept in memory. This depends on the ability of the frontal lobe to automatically select memories of past situations related to the present ones and alert the person to the likely consequences of several distinct decision scenarios—according to experience, that is, memory. 5 Crucially, the third module is to move beyond the simple anticipation of consequences in the *intellectual* plane of *knowing* what is best, and bring into the equation the affective *feeling* plane, anticipating negatively-valenced *feelings* (sadness, shame, embarrassment, guilt) that will be felt with negative consequences in the future of inadequate decisions taken now. We know for a long time that without such emotional contribution, currently understood as dependent upon fronto-insular sensory cortex connectivity, decision making may be disastrous. 6 7 8 Finally, the fourth module involves the use of time in one's favor, both activating behavior to get tasks initiated (i.e., not procrastinating) and correctly estimating the time needed to perform tasks, thus getting things done and meeting deadlines.

These difficulties often seen in adults with ADHD translate into not functioning well in the social context—or, in a reduced capacity to fulfill social demands. Practical symptoms include not meeting deadlines, a low level of persistence leaving tasks incomplete, delays in initiating relevant activities, disorganization of priorities, impulsive decision making and the need to constantly excuse oneself for not having been able to integrate intentions with action, highlighting the hiatus between theory and practice. 1 Furthermore, persons with ADHD often present emotional dysregulation, dealing with frustrations with irritation and explosive behaviors, which lead to negative consequences.

Thus, the treatment of adults with ADHD seeks to offer an opportunity to better organize their lives and affects—and, in so doing, to be more effective socially. As we will see, successful therapeutic efficacy with such an ambitious goal will demand a combination of approaches, beginning with psychosocial education and involving the judicious use of medication and psychological support.

Two additional points should be touched upon to explain the multilayered approach. One is that until not very long ago, starting an article on ADHD with a brief promenade through the executive functions needed for a reasonably successful adult life would not make much sense. Attention deficit hyperactivity disorder was then seen as a childhood disorder and, in the fourth edition of the DSM, published in 1994 and anticipated to last (as it did) for two decades, the symptoms needed for diagnosis were based in the childhood presentation. 9 That was partially corrected in DSM V, 10 published 11 years ago, although still in a tentative fashion, because it is missing important symptoms. The other point is that the interest in ADHD skyrocketed in the last three decades, with many important advances in pathophysiology, prompted by exciting genetic and neuroimaging data. 11 12 13 14 15 Therefore, although my main goal is to make the case that adult ADHD is in fact a highly prevalent disorder of inattention to the future, I will start with a necessary revisitation of the basics of ADHD epidemiology and the core symptoms for a DSM-based diagnosis—and that is what I denominate the first layer. In the other, deeper layers, I will then integrate pathophysiological aspects with treatment strategies.

## LAYER 1: EPIDEMIOLOGICAL AND CLINICAL CONSIDERATIONS AT THE SURFACE

Attention-deficit/hyperactivity disorder affects around 5% of the pediatric population, and around half of these kids do not outgrow it, leading to a prevalence of ∼ 2 to 2.5% of adults with ADHD. As in other mental disorders, clinical manifestations must now be assessed in the light of the DSM-V, that sets a minimum symptom threshold to define the diagnosis. Thus, there is DSM-V-defined ADHD and subthreshold ADHD, with a combined prevalence 4 to 5%. 16 17

The risk of the disorder in parents and siblings of children with ADHD is increased 2 to 8 times in comparison to the general population; 11 , therefore, genes certainly influence susceptibility, particularly polymorphisms in the dopamine transporter (DAT1, SLC6A3) and the dopamine 4 receptor genes (DRD4). 12 13 This suggests a complex etiology in which the disorder is caused by the interaction between these polymorphisms with environmental risk factors, such as prematurity, low birth weight, and several prepregnancy and pregnancy factors such as maternal smoking, obesity, and hypertensive complications. 18

Symptoms in childhood have been extensively studied and comprise the DSM-based diagnostic criteria. 9 10 Most readers will be familiar with the clustering of symptoms related to inattention, easy distractibility, lack of concentration, impulsive behavior, and hyperactivity. For some time, neurologists and psychiatrists struggled with how to translate such symptoms ‘crafted’ to ADHD in children into the complex manifestations in adults. The DSM-V has somewhat improved this situation, with criteria more suitable for the adult population: inattention now includes problems remaining focused on a task for long periods, as well as difficulties in organizing activities, prioritizing and completing tasks 10 .

Interestingly, forgetfulness is also a common complaint. 1 I say to patients that attention is memory's waiting room. If an information is received while the person is not paying attention to its source, chances are that it will be lost. It is a universal experience: if one leaves an object (the keys, the cell phone) in any spot at home or in the office while the attention is somewhere else, it takes much longer to locate the object. Extrapolate that to most daily activities and it is understandable why people with ADHD complain of forgetfulness. Moreover, impulsivity in adult ADHD has potential dire consequences at home, in the streets or at work, many times leading to relationship problems, quitting jobs, or driving mistakes. 1

The Paul Wender group, from Utah, was the first to draw attention to the emotional dysregulation presented by many adults with ADHD. 19 Mood lability, irritability, anger outbursts, low frustration tolerance, and motivational deficits are commonly seen, and often pose problems related to the differential diagnosis between ADHD and other psychiatric disorders or raise questions about whether a comorbid disorder is present. 20 21 22 23 This is no small issue, because management is quite different should one consider that these abnormalities are due to comorbid bipolar disorder or major depression, or only to ADHD. What is meant by emotional *dysregulation* is an inability to manage uncomfortable emotions and move on with life when distressed—instead of being incapacitated by the distress. Emotional lability and negative emotional responses, two dimensions of emotional dysregulation, correlate closely with ADHD symptoms severity. 21

Thus, in summary, the basic symptoms relevant for an ADHD diagnosis in adults do not depart very much from those in childhood. However, as I will be discussing in layers two and three, the main or defining features in adults are the many facets of executive dysfunction, that is, difficulties to make life work, that is, to define what one wants to achieve at any time, from the simplest to more sophisticated goals; to design a strategy to pursue such goals and to persevere, inhibiting interferences and sustaining the necessary motivation until conclusion. Recently, Ustun et al. proposed a screening scale with high sensitivity and specificity for adult ADHD diagnosis—the Optimal RiskSLIM DSM-5 ASRS Screening Scale, based upon the frequency of the following 6 manifestations 23 :

- How often do you have difficulty concentrating on what people say to you, even when they are speaking to you directly?
- How often do you leave your seat in meetings or other situations in which you are expected to remain seated?
- How often do you have difficulty unwinding and relaxing when you have time for yourself?
- When you are in a conversation, how often do you find yourself finishing the sentences of the people you are talking to before they can finish them themselves?
- How often do you put things off until the last minute?
- How often do you depend on others to keep your life in order and attend to details?

Such questions are useful, as the diagnosis of ADHD in adults is often not easy. Two quite distinct issues converge in posing difficulties: on the one hand, many of the psychiatric disorders in the differential diagnosis are also commonly comorbid with ADHD, such as mood, anxiety, and substance use disorders (SUDs). On the other hand, some symptoms of adult ADHD, both the core DSM and the more refined manifestations of executive dysfunction are present in every person to some degree. Everyone is occasionally distracted by an interfering stimulus, makes some impulsive decision, procrastinates, underestimates time, etc. Therefore, the definitive diagnosis is based upon severity, number, and functional impact of symptoms—not the isolated presence of one or two.

Regarding the resemblance to psychiatric disorders, one should keep in mind some of the key differentiating features that may be confused (or associated) with ADHD and, thus, need specific treatment. For instance, depression and mania can be distinguished from ADHD in adults by a typically later onset and episodic course. Furthermore, mania usually presents with a combination of elated mood, rapid cycling, grandiosity, and increased sexuality, all suggesting a bipolar disorder. 25 Another common entity in the differential diagnosis/comorbid continuum—anxiety disorders—usually shows distraction in the presence of florid anxiety symptoms or in situations that hijack attention, such as obsessional thinking in obsessive-compulsive disorder or fear of failure in generalized anxiety disorder. Poor attention, distractibility, impulsivity, and hyperactivity are shared by ADHD and SUDs, although in the latter, symptoms are usually temporally related to the use of the substance(s). 22 23

The universality in the general population of some symptoms of distractibility, poor concentration, forgetfulness, impulsive decisions, and occasional procrastination must also be kept in the mind of the professional, particularly because pari pasu with the growing *scientific* interest in ADHD, there has been a growing *popular* interest in the disorder. As self-rating scales became easily available online, it is not uncommon for people to self-diagnose ADHD. Thus, the clinician must take advantage of the neurodevelopmental perspective of ADHD and inquire about symptoms throughout the life spam, even allowing for inconsistent recollection of childhood manifestations, as well as paying attention to aspects of the emotional and occupational life of the patient (e.g., burnout) that may account for what looks like ADHD symptoms.

### The “downward schooling spiral” and persistence of ADHD in adult life

Many adults with ADHD will report a tortuous schooling trajectory that I denominate ‘the downward schooling spiral.” and paying attention to this may facilitate diagnostic suspicion of ADHD. The premise of this downward spiral is that healthy parents, irrespective of social class, will try to provide the best possible schooling for their children within their means. However, poor school performance (due to a combination of inattention, difficulties to concentrate and complete tasks, impulsivity/hyperactivity, and/or comorbidity with a learning disorder) leads to low self-esteem and recurrent failures, prompting a change to another, usually less demanding school. Because symptoms continue, leading to further failures, not uncommonly, these people eventually move to a third school, further down the spiral; this third school is even less demanding and more prone to accommodate other people with similar difficulties. Many eventually drop out or finish school through some alternative means, which—combined with other ADHD manifestations—often limit academic and professional achievements.

Finally, this first layer must address the universal question of parents and patients on whether childhood ADHD symptoms will eventually subside (and treatment may be stopped). Multivariate analyses of longitudinally collected data have shown that the most important childhood predictors of adult ADHD symptom persistence are initial ADHD symptom severity, comorbidities, particularly with oppositional defiant disorder and social phobia, and parental mental health problems. 26 27 Overall, between 40 and 60 percent of children with ADHD will eventually have ADHD-related problems in adulthood. 28

## LAYER 2: A DEEPER UNDERSTANDING OF COGNITIVE MECHANISMS OF ADHD IN ADULTS, AND THEIR ANATOMICAL BASES

In 1993, the Neurologic Clinics of North America, published a fascicle on Behavioral Neurology, in which an article by Martha Denckla drew attention to residual symptoms of childhood developmental disorders in adults, particularly focusing on dyslexia and ADHD. 28 Although, per se, that was not new, in practice, the topic was systematically neglected, and that publication had the merit to kindle the interest of neurologists and psychologists for people with subtle, albeit relevant, alterations in daily functioning. Until that point, ADHD was essentially seen as a psychiatric disorder, but even psychiatrists often failed to reach a diagnosis in persons with difficulties to manage their lives without other major symptoms that fit classic psychiatric syndromes—or, alternatively, (and despite subsyndromic presentations), would attempt to make these people fit into formal diagnosis, such as mood or anxiety disorders. Interestingly, around the same time, a book was published with the enticing title *Shadow Syndromes* , 29 describing people with subtle alterations occupying the frontier between symptoms purely attributable to psychological contexts and *forme frustre* of classical psychiatric disorders. They had as common denominator a recurrent sabotage of daily functioning, leading to frustrations and difficulties in moving on with their lives—and the possibility that many had the then poorly known adult form of ADHD was discussed by the authors.

In this second layer, I will attempt a more profound analysis of what really happens with adults who have ADHD, and that will set the stage for discussing treatment strategies. Because this review focuses on ADHD in adults, it makes sense to begin with what is expected of an adult.

### What is expected of an adult?

Attention-deficit/hyperactivity disorder in adults has assumed progressive relevance to explain difficulties in daily life, and to understand these difficulties, the starting point is what is expected of an adult concerning life management. Despite socio-occupational changes brought about by technology, it is still expected that adults (most of the time) honor their commitments, prioritize activities and dedicate themselves to complete tasks, organize time, control impulses, contextualize emotions, utilize memory to orient decisions, and follow the necessary steps to reach goals. Because adhering to each of these basic things in daily life translates into successful or failed outcomes in whatever activities or ideas they are employed—and these outcomes are verified in a (variable) future time—in simpler words, what is expected from an adult is the monitoring of behavior considering future consequences. 1 In its adult presentation, ADHD interferes with such flow, and its main manifestations can be seen as the inverse image of what is expected from an adult. These people have difficulties to keep concentration for the duration needed to read reports or complete tasks, lose a lot of time to get organized and activate their behavior (i.e., start) toward doing what they should do. They often scramble priorities and procrastinate urgent or relevant tasks (that demand more attention, effort, and bring less pleasure) in favor of easier and more stimulating activities. This is compounded by problems with impulse control and managing time, often with underestimation of the time needed to complete tasks, leading both to procrastination and finishing tasks at the last minute—with less quality than would be the case in more favorable circumstances. In other words, these people do not do what must be done at a particular point in time, leading to negative consequences (in the future) and the recurrent need to excuse oneself. Moreover, people with ADHD complain of difficulties with memory and frequently forget appointments and combinations—exactly because inattentive to the issue at hand, with a tendency to focus elsewhere when the memory of simple things in daily life is being formed.

### Nudging toward the present

The growing interest in ADHD in adults has led to several interesting scientific advances, contributing to the comprehension of what really represents this disorder and its underlying cognitive mechanisms. Thus, over the years, several cognitive axes have emerged to explain the bases of the (dys)-functioning of these individuals, which add to the (dys)-executive context described above. One is that ADHD would represent difficulties to delay gratification, *nudging* the person to intrinsically rewarding activities in the present, thus failing to put effort in activities for which reward will come in the future, following the conclusion of a difficult task, for instance. 31 This perspective supports the view of ADHD as a disorder of excessively anchoring decisions in the present instead of in the future (consequences).

A modern view of the reward system posits that this system—centered around the connections of the nucleus accumbens with the ventral tegmental area of the mesencephalon and the prefrontal cortex—has the *dual function* of giving the *drive* to explore the environment to obtain rewards and *signal* when such rewards are obtained. 32 Obviously, the chances of getting a reward are greater when one goes after it; thus, activating the reward system when there is a *perspective of obtaining a reward* is crucial to the drive and effort to go explore the environment and get it.

This notion has received support from neurobiological data suggesting that anatomical structures of the reward system of the brain in adults with ADHD activate *less* with the perspective of a future reward and *more* with the reward itself. 32 In contrast, subjects in the control group showed the opposite, that is, greater activation of reward structures with the *perspective of getting a reward (in the future)* . This finding is crucial to the hypothesis I put forward, that ADHD in adults represents an *inattention to the future* .

Interestingly, the delayed gratification insensitivity theory connects very well with still another neuropsychological mechanistic perspective, posing that people with ADHD have energetic difficulties related to weak integration between subcortical structures, related to arousal, and cortical regions, engaged in executive function—that is, the neural integration underlying what we know by effort 33 . According to this theory, it would be more difficult for these people to get activated and persevere for the time needed to complete tasks or pursue a strategy to achieve a goal. Interestingly, an effort—or, in its extreme, a sacrifice—only makes sense if one has a future goal in mind and succeeds in approximating that future to the present, when the effort must be made.

The notion that ADHD represents abnormalities in neurodevelopment makes it difficult to bridge the problems with concentration or paying attention (in a task, a class, a movie)—the essence of ADHD in children—with the complex manifestations of executive dysfunction in adults. That is the reason for the construct *inattention to the future,* proposed to bridge childhood and adult ADHD symptoms. Under this perspective, difficulties with the representation of the future in the present is the essence of inattention in ADHD—and I posit, the common denominator for the 3 prevailing neuropsychological hypotheses underlying the disorder:

- the executive dysfunction;
- the dependence upon immediate rewards;
- imbalances in the energy distribution to activate behavior.

The present is inevitably sandwiched between the past and the future. 34 35 Everything we do or do not do—including the decision to activate our behavior (or not), to perform laborious tasks instead of pleasurable ones, and to control impulses (avoiding acts that could prove harmful in the middle/long-term)—in brief, the whole context of behaviors *in the present* , takes into account both past experiences in similar contexts and future scenarios that must be anticipated as the most likely outcomes of each possible decision. 36 Thus, past/present/future are intricately connected, and the reason behind the disconnection between the future and the present is that *inattention to the* *future* is the central problem of adults with ADHD.

### The future does not exist: the neurobiology of approximating the future to the present

In concrete terms, the future does not exist. We simply have no means to know what is going to happen days, weeks, months or years ahead; thus, any notion of the future is essentially abstract. It follows that the notion of future consequences of our acts is also somewhat abstract. On the other hand, despite such abstraction, our decisions, plans, resolutions, and sacrifices are always based upon the future (i.e., upon something that does not exist). Therefore, an inevitable question is: how does the brain allow such a thing? (i.e., to organize our behaviors having in mind something that does not exist)?

The brain has circuits that represent the future—that is, they approximate the future to the present and, in so doing, create concrete scenarios of the possible consequences of our decisions. 2 35 37 These brain circuits are centered in the multimodal associative cortex of prefrontal regions and its subcortical connections. The prefrontal cortex associates and synthetizes information originated in distinct cortical regions which converge upon it and are integrated as a unique experience. 37 A good analogy is a 5-letter word, each letter originated in a distinct brain region and connected to the prefrontal cortex. The latter assembles the letters and forms the word. Each letter, in isolation, has its own relevance (e.g., an olfactory stimulus, an idea, a will, a visual perception, a memory, an emotion), yet assumes even greater relevance for decision making once integrated with the other letters in a full word. Seen from another perspective, the prefrontal cortex functions as a kind of super-hub, receiving information from areas processing sensory modalities, memory regions, and structures involved in emotional processing—integrating and synthesizing these sets of information, remembrances, and perceptions into action motor programs. These frontal lobe structures receive a polymodal set of information, originating from all corners of the brain and generated from both personal experiences and cultural transmission, creating a databank of ‘good and bad’ experiences. Each new experience or decision is analyzed from this databank perspective and generates expectations about future outcomes (positive or negative). More specifically, from this experience databank, prefrontal regions form representations of actions to harvest rewards and avoid punishment. 38 Hence, when we decide to not eat chocolate cake today to reduce the levels of cholesterol or be slimmer six months later, such perspective is entered into the “experience databank”, analyzed according to what has happened in other similar situations or what the culture transmits about the impact of chocolate cake on cholesterol levels and weight, and, most likely, will confirm that avoiding it is worth the effort (sacrifice), even if the craving and the cake are very much real and concrete in the present, and the impact upon weight and metabolism are abstract and will be seen/felt only in the long term (i.e., in the future). 39 The idea I propose is that ADHD interferes with the capacity to integrate information in the prefrontal cortex and, thus, use the “experience databank” to guide decisions. This leads to what I call *inattention to the future* , that is, to the consequences of decisions or behaviors in the middle and long term. However, such inattention has a degree of subtlety, manifested by the discrepancy between the *theoretical, intellectual knowledge* of what, when, and with which level of effort a given thing must be done on the one hand and the allocation of the necessary energy/effort to pragmatically activate behavior and put this into *practice* . This discrepancy is at the base of constant regrets of ADHD patients, who profess they know/knew they should have done things in a specific way but have not done that. In other words, knowing what must be done *in theory* is very different from doing that *in practice* , as we all know, and these patients know better than most people.

It is likely that maturational abnormalities in prefrontal circuits fail to adequately signal reward/punishment perspectives in the future, as supported by neuroimaging studies in patients with ADHD showing maturational changes in circuits involving the prefrontal multimodal associative cortex. 40 41 A classical longitudinal neuroimaging study comparing typical subjects with ADHD children and adolescents showed a significant maturational lag in frontal lobe structures in those with ADHD, 40 and more recent studies in young adults not only confirmed those findings but advanced abnormalities in white matter tracts connecting the prefrontal cortex with other cortical regions, particularly the superior longitudinal fasciculus and cortico-limbic circuits. 41

### “Subjectively I am a rich man, objectively I am a beggar”: the less visible yet crucial role of intermediate processes in achieving goals

This sentence, quoted from a man I saw many years ago, is a good synthesis of the inattention to the future in adult ADHD. He was very good at generating ideas for businesses and has worked as an entrepreneur in several companies. All eventually went bankrupt, and there was a common theme to all: initially, business blossomed, but progressively failed because of his difficulties to pay attention to simple (bothersome, yet important) issues such as keeping account flowcharts, respect payment deadlines, regulate inventory, and avoid impulsive decisions. His administrative style eventually amounted to a level of recklessness that was incompatible with keeping the business, yet because things always started very well, he could not understand why things turned out that badly every time. His story emphasizes the difficulty of anchoring in the future what I denominate ‘the laborious intermediate processes’ of anything we plan to achieve: those steps that, in themselves, are laborious and seem of little relevance, yet must be done to bridge a good idea, or a good intention to a positive outcome in the end. People with ADHD often have very good ideas, and the drive and the stamina to initiate or engage in projects, companies, tasks, etc, yet have a tendency to allow interferences, divert attention and lose the energy “long the way—that is, along the laborious intermediate processes—eventually losing focus and failing to achieve the planned goals.

## LAYER 3: EXPLORING THE DEPTH: NEW CONCEPTS, DISTINCT PROFILES, AND THE AMBITIOUS TREATMENT OF ADHD IN ADULTS

### New concepts about ADHD in adults: the eruption of the geyser

New concepts are like geyser eruptions: in this natural phenomenon, the underground water has always been there, yet needed time to reach the necessary boiling temperature and erupt. Similarly, clinical experience and research data slowly accumulates until reaching a boiling point, that is, a point in which new knowledge and concepts are generated and become incorporated into the mainstream. This is a good analogy to the recent perspective that, in some patients, ADHD may present only in late adolescence and adulthood, challenging the longstanding concept that the diagnosis needs a history of childhood ADHD. 42 43 After decades hesitating in making the diagnosis of ADHD in adults with unequivocal symptoms, yet without obvious or inconsistent history of ADHD symptoms in childhood, several research groups collected longitudinal data to show that many patients only present with significant symptoms in adulthood and did not fulfil diagnostic criteria in childhood. 42 43 In one of these cohorts, with more than 1,000 individuals followed up until the age of 40, there was a prevalence of ADHD in childhood of 6% and of 3% in adults. Interestingly, only 15% of those with childhood ADHD retained that diagnosis in adulthood and only 10% of those with a diagnosis in adulthood had suprathreshold symptoms in childhood. Furthermore, an elevated percentage of adults with ADHD did not have comorbidities that could confound the diagnosis. These studies have been recently compiled and led to the proposition that there are several possible symptom trajectories of ADHD through life. 44 Moreover, they have opened a fruitful debate on possible causes for the emergence of ADHD in adulthood—even continuing to acknowledge that ADHD is indeed a developmental disorder. Thus, although it is important to track symptoms since childhood, with the inherent recall biases, the possibility that the disorder may have been only latent during childhood, without significant symptoms at that stage, yet emerge as a full-blown disorder with the increase in demands during adolescence and adulthood should be considered. Clearly, the childhood universe in very different from the adult one: childhood demands are centered around family and school, whereas adults must deal with work, a broader social level of demands, affective relationships, financial responsibilities, access to substances, and the need to make plans to manage life. Thus, a fertile psychosocial context may prompt the emergence in adulthood of previously latent ADHD symptoms.

A few years ago, Shaw et al. studied structural indexes of cortical development in a cohort of normal children and showed that the pace of cortical maturation correlated with symptoms of inattention, hyperactivity, and impulsivity, and that adolescents could both have subthreshold number of ADHD symptoms, yet suffer from negative functional impact. 44 In other words, variable levels of cortical (dys)maturation underlined these symptoms, and perhaps more florid symptoms could eventually appear as demands increased in adulthood. Therefore, it is possible to envisage four intersecting axes impacting upon the occurrence, timing, and trajectory of ADHD symptoms through life:

- a neurobiological axis, which determines the pace of cortical maturation;
- the level of demands that life brings;
- the kind of psychosocial support granted through life and, lastly,
- a history of brain insults and comorbidities.

A combination of these factors may determine if, and when, symptoms emerge and with which severity—even if there was already a biological tendency linked to neurodevelopment.

#### *The pillars of diagnosis in light of the novel concept that ADHD symptoms may start in adulthood*

Adult ADHD diagnosis rests upon three main pillars: initially, whether symptoms fulfil DSM-V criteria, according to either a non-structured interview or some validated instrument. Symptom listing of DSM-V incorporates elements more appropriate for adolescents and adults, translating into the executive dysfunction domain purely inattentional manifestations in childhood. One good example is the item *get easily distracted* (in childhood), that in adults is replaced by *deviating the focus of attention to things not related to the task at hand* . Moreover, acknowledging that symptoms may reduce in intensity over the years, official criteria has now reduced the diagnosis threshold to only 5 (instead of 6) of 9 possible symptoms of inattention and/or hyperactivity-impulsivity.

The second pillar is the differential diagnosis or the identification of comorbidities with at least three frequent psychiatric conditions: depression, bipolar disorder, and anxiety. This demands a psychiatric evaluation, although many adults with ADHD present to the neurologist already being seen or having been seen by a psychiatrist. Many symptoms of adult ADHD, whether related to executive dysfunction or the more canonical inattention, poor concentration, easy distractibility, or impulsivity, may be part of other primary psychiatric disorders, that may co-occur with or be independent from ADHD. Furthermore, other aspects in the life of the person may lead to these same symptoms, including emotional difficulties related to losses or family conflicts, the abuse of sedative medications, alcohol or marijuana, sleep disorders, and burnout. Therefore, when considering the differential diagnosis or the possibility of comorbidities, the neurologist should realize that restlessness may be a symptom of ADHD, but also of an anxiety disorder or of emotional difficulties. Likewise, emotional decontrol may be a symptom of ADHD, but also of an alternative or comorbid bipolar, personality, or substance use disorder, as well as an expression of emotional difficulties. Thus, because the diagnosis is essentially clinical, a detailed interview is imperative.

The third pillar—which helps to clarify the second—is the delineation of the longitudinality of symptoms. Despite the new conceptualization of adult ADHD as a disorder that may manifest only in adulthood, investigating the symptom trajectory during life is important and helps diagnosis in many patients—as data has shown that in three fourths of patients, symptoms do start in childhood. 46 Thus, although presentation may differ at different time points, ADHD symptoms are often present throughout life, and even if recall bias is a problem, solid pieces of evidence should be sought in the past: a history of the downward schooling spiral (see above), a negligent style of studying, incomplete tasks or excessive time to complete assignments during childhood and adolescence due to easy distractibility, a record of poor book reading, the level of effort to move on to next levels along the ladder (find the first job, going into college), and the overall alignment or discrepancy between the intellectual level of the patient and what has been achieved in life.

### Two distinct profiles of adults with ADHD: from failure to effortful success

The negative impact of the symptoms of inattention to the future is the major problem of adults with ADHD, which goes way beyond not paying attention to a movie, a book, or the placement of a document needed at a particular moment. Longitudinal studies show that these people endure significant professional, affective, and family difficulties, have more problems with the law and a tendency to abuse alcohol or drug. 1 However, it is important to recognize two distinct profiles of adults with ADHD, according to the degree of discrepancy between the intellectual capacity of the person and the level of satisfaction (personal, professional, affective) reached in life. 47 One profile is that of those individuals in whom ADHD symptoms were/are so pervasive in life that they have fully failed trajectories. This is a sad situation, because many would have had very good chances of professional and affective success, because of their good intellectual capacity. Their trademark is an impressive discrepancy between intelligence and opportunities on the one hand and the meagre practical achievements in life on the other—demonstrating the poor outcome associated with unidentified and untreated ADHD.

The other profile characterizes individuals who are reasonably successful despite their ADHD, because they mastered compensation strategies—albeit often with considerable effort and suffering. These are individuals that succeed at least partially in realizing their potential, however at the cost of constant self-policing in order to do what must be done; otherwise, should they just allow life to flow, they would fail. In other words, these individuals need constant monitoring of their situation, actively combating interferences, getting help to keep appointments, make copious notes of everything, etc., so that things are done and appointments are kept. This level of effort has a cost and lead to exhaustion and the need to work longer hours, often into the night, leaving aside other aspects of personal and family life, as if all energy needs to be allocated to compensate for the ADHD symptoms. 47 Furthermore, because poor organization and tendency to procrastinate and scramble priorities are features intrinsic to ADHD, these people develop the strategy of leaving things to the last minute.

#### *Can a medication change a life?*

Simply communicating the diagnosis and prescribing a medication is usually ineffective to treat ADHD in adults. The neurologist needs emotional intelligence to explain the problem in detail, empathizing the difficulties and demystifying symptoms that have been the frustration trademark of that person throughout their life. Explaining in detail what happens in the brain, particularly the failure of neurobiological mechanisms to focus attention, finding salience (i.e., realizing the importance of what has to be done), and blocking parallel interference already alleviates the tension of someone who has been constantly blamed and seen as incompetent. This sets the stage for communicating to the patient what is to be expected from medication. There must be a bidirectional relationship between psychosocial education and medication, without which medication will not be very effective.

Unfortunately, the effect of stimulants in adults with ADHD used to address executive dysfunction—and the many negative consequences in the professional, academic, financial, social, and family spheres—is far from obvious. Based on DSM-IV or V-derived scales of symptom modification, most studies target the core symptoms of inattention, lack of concentration, hyperactivity, and impulsivity. 49 50 In these studies, methylphenidate, lisdexamphetamine, and atomoxetine, which act primarily upon cortico-subcortical dopaminergic and noradrenergic circuits, have shown efficacy for tasks such as paying attention at meetings and conferences, reading, and preparing documents, that is, activities that require concentration and in which performance is better when people are less distracted. 51 However, these studies did not address other major issues; therefore, the impact of medication on most manifestations of executive dysfunction, such as impulsive decision making, procrastination, and scrambling of priorities, for instance, is poorly known.

I suggest assessing the effect of psychostimulants at two levels, according to the degree of executive dysfunction and the use of compensatory strategies:

- improvement in the capacity of defining goals, establish strategies, and follow these strategies to reach goals, and
- reduction of the need for constant and exhaustive self-policing to compensate for symptoms of inattention, procrastination, and disorganization.

In the first level, it should be observed whether psychostimulants are helping to concentrate on some goals and to anticipate the positive consequences of reaching those goals. Although well-designed studies to this end are lacking and the available evidence is far from clear, 52 some patients do report being more focused on their goals and mustering more energy to achieve them. This is important to manage executive dysfunction, because a significant number of patients cannot define objectives or devise a strategy that would commit them to apply the necessary effort to harvest future rewards. Based upon the cognitive notion that executive functions, in essence, allow us to act today anchored in the future (consequences), the possibility to anticipate consequences is necessary to organize behavior in the present. Thus, the report of some patients that medication helps to focus on future goals is already important.

Moreover, I use the metaphor of the jigsaw puzzle to explain what I consider the role of psychostimulants in the life of adult patients with ADHD. Imagine the life of these people as scrambled pieces of a jigsaw puzzle, with no hint of what image should be formed once pieces are correctly assembled. In this metaphor, stimulants work as a *set of key pieces* that, once assembled, allow delineation of what figure shall be formed, that can be recognized even without several missing pieces. Identifying the figure would be akin to define one or two key objectives in life and some basic steps toward them—an already significant step for people that often have no clue on how to start a sequence of intermediate processes to achieving goals in life. Note that the picture is not complete with these key pieces—that is, objectives are, of course, not immediately delineated or reached simply by using medication—but it is simpler to fit the remaining pieces when one has a clue of what image should be formed.

Another therapeutic approach, complemented by much needed psychological support (usually cognitive-behavior therapy 53 54 ) is to emphasize that psychostimulants will be more likely to make a difference if integrated into three behavioral elements summarized by the acronym MOG: motivation, occupation, goals. This must be explained to the patient: *motivation* is a necessary first step to find ways to move on with life, organize behavior, and put effort into whichever activities are at hand. Such *motivation* , in its turn, should be complemented by an *occupation* , in which motivation will be enacted. It does not suffice to be motivated to improve or reach a goal, an *occupation* where improvements may be assessed is needed, whether a course, a new job, a period of study for an exam, etc. Finally, in the context of this *occupation* for which the patient is *motivated* , there must be clear and achievable *goals* . This goal-related outcome is, of course, based upon the *motivation* and *occupation* . As such, goals should be simple and involve engaging in the intermediate processes, which surely increases the chances to achieve *goals.* For instance, the goal of a given patient may be to activate behavior to concentrate in the study of didactic material for a test for 2 hours every evening. Note that the *goal* is not to succeed in the text, which depends on several other variables unrelated to the effort of the patient, but to do what should be done to have real chances to succeed (for instance, dedicate a predetermined number of hours studying for the test). Hence, psychostimulants may be very important to allow the patient to go after the goals, reversing the noxious cycle of not doing what must be done to have real chances to succeed (and then recurrently failing), improving self-esteem, and avoiding reinforcing the feeling of incompetence reported by many adults with ADHD.

The other effect of psychostimulant treatment of adults with ADHD applies to those reasonably well succeeded professionally because of compensation strategies. Because strategies aimed at reducing the negative impact of ADHD symptoms demand effort and constant self-policing, they often lead to exhaustion, and allocation of energy that negatively impacts upon other aspects of personal and family life. In these patients, stimulant medication reduces the need to self-police and to be constantly monitoring behavior to avoid making mistakes. 47

#### *Which medications and how to use them*

There is no clear advantage of one versus another stimulant medication, and there are no head-to-head comparisons of efficacy between stimulants and atomoxetine, a non-stimulant, adrenergic drug. Stimulants tend to act more rapidly while atomoxetine may take longer, up to a month or two, including the time needed to fine tune the correct dose for each patient. 55 Furthermore, although direct comparisons are not available, meta-analyses have shown that effect sizes in short-term trials of adult ADHD are greater for stimulants compared with nonstimulant medications. 56 57 However, some patients do report improvements with both and better tolerability with atomoxetine.

Among stimulants, it is not clear which one works best, whether it is lisdexamphetamine or methylphenidate. In adults, long-acting preparations are usually preferred, although, as time passes, some patients identify specific moments in their days when a stimulant is needed to improve attention and concentration, and, thus, prefer short-acting preparations. In a network meta-analysis of short-term effects (3 months) including more than 8,000 adults with ADHD, individuals treated with amphetamines showed greater improvement on clinician-rating scales of overall ADHD symptoms than methylphenidate, although the amphetamine used in most studies (mixed salts dextro-amphetamine/amphetamine, branded Adderall [Teva Pharmaceuticals, Cambridge, MA, USA] in the US) is not available currently in many countries, including Brazil. However, amphetamines were associated with higher risk of treatment ending early as the result of adverse events. 57 Should lisdexamphetamine be the stimulant of choice, it is important to titrate the dose, usually starting with less than 30 mg a day (the smallest pill available in some countries) to reduce adrenergic side effects.

### Other aspects related to pharmacological treatment of adult ADHD: cardiovascular risk and management in the presence of comorbidities

The possibility of adverse cardiovascular effects due to psychostimulants with adrenergic function in adults has been studied, with mixed results. While some populational studies show no association or increased risk, others suggest there may be a mild increase in the risk of cardiovascular events. 57 58 There is, however, a consensus that before prescribing a psychostimulant, a thorough personal and family history for cardiac disorders and risk factors should be taken and, when indicated, a cardiologic assessment performed. It is important to mention that only rarely there is a formal contraindication for these medications, although in some cases psychostimulants must be used with caution. With this being said, very recent data suggest that mortality decreases in adults with ADHD on pharmacological treatment, which is an important observation. 59

Although somewhat outside the scope of this review, it should be mentioned that given the high prevalence of psychiatric comorbidities in adults with ADHD, a high level of suspicion is in order. When a comorbidity is diagnosed, the neurologist and the psychiatrist must determine whether:

- ADHD should be treated first,
- both entities should be treated concomitantly from the start – or else,
- the comorbid disorder should be treated first, and only after stabilization the ADHD medication should be safely prescribed.

I have mentioned above the main psychiatric comorbidities and one or two aspects related to their diagnosis and differential diagnosis with ADHD. When there is comorbid anxiety or depression, a reasonable strategy is to start treating ADHD, as many patients will show improvement of both entities with psychostimulants or atomoxetine. When there is SUD, it is important to concomitantly target ADHD symptoms with stimulants or atomoxetine and approaches (pharmacological or otherwise) to the SUD. Finally, when there is comorbidity with bipolar disorder, the risk of inducing a manic state with stimulants implies that mood stabilization should be the first goal with whatever pharmacological strategy, and only then should ADHD medications be prescribed.

### A final word: ADHD in adults is perhaps the most prevalent frontal lobe disorder in humans

Most neuropsychiatric disorders have a denomination that immediately connects with their main manifestations. Even if the diagnosis may occasionally be difficult, it is immediately understood what is meant by depression, anxiety, bipolar disorder, obsessive-compulsive disorder, epilepsy, or dementia, to name a few. For many years I struggled with the link between the name attention deficit/hyperactivity disorder in adults and the large number of dysexecutive, impulsive, and procrastinating symptoms. Integrating the rich clinical research of ADHD with the well-known connection between executive functions, impulse control, and behavior activation as frontal lobe functions, I came to the understanding proposed here that the attention deficit in the ADHD denomination is an *inattention to the future* —and thus, as I suggest in the text, ADHD is, above all, a frontal lobe disorder, perhaps the most prevalent (because developmental and not related to trauma) frontal lobe disorder in humans. I hope this perspective will help to identify more people suffering from this serious disorder and foster innovative treatments to mitigate their suffering.
